# A systematic review and meta-analysis of the role of sugar-free chewing gum on *Streptococcus mutans*

**DOI:** 10.1186/s12903-021-01517-z

**Published:** 2021-04-29

**Authors:** Melanie Nasseripour, Jonathon Timothy Newton, Fiona Warburton, Oluwatunmise Awojobi, Sonya Di Giorgio, Jennifer Elizabeth Gallagher, Avijit Banerjee

**Affiliations:** Faculty of Dentistry, Oral and Craniofacial Sciences, King’s College London, Floor 26, Guy’s Dental Hospital, Great Maze Pond, London, SE1 9RT UK

**Keywords:** Prevention, Polyols, Xylitol, Children, Adults, Clinical trials, Oral bacteria, *Streptococcus mutans*

## Abstract

**Background:**

Preventive strategies targeting *Streptococcus mutans* may be effective in reducing the global burden of caries. The aim of the current systematic review of published literature was to determine the difference in level of *Streptococcus mutans* in adults and children who chew sugar-free gum (SFG), compared with those who did not chew gum, who chewed a control gum or received alternatives such as probiotics or fluoride varnish.

**Methods:**

Systematic review (PROSPERO registration No. CRD42018094676) of controlled trials with adult and child participants where chewing of SFG was the main intervention. Databases searched (1 Jan 1946 to 31 August 2020): MEDLINE, EMBASE, PsycINFO, Scopus, Web of Science, Allied and Complimentary Medicine Database, Cochrane Central Register of Controlled Trials (CENTRAL), Open Grey, PROSPERO and the Cochrane library of systematic reviews. ‘Search terms included Medical Subject Headings, and free text to cover the following range of constructs: chewing gum, sugar free, oral health, caries, xerostomia, periodontal disease. Data extraction and Risk of Bias assessment was undertaken by three researchers using a modified version of the Cochrane RoB tool (version 1). Data synthesis was conducted using meta-analysis in STATA.

**Results:**

Thirteen studies of SFG with micro-organisms as outcomes were identified. The use of SFG significantly reduced the load of *Streptococcus mutans* (effect size − 0.42; 95% CI − 0.60 to − 0.25) compared to all controls. In seven of the 13 studies the confidence intervals of the effect size estimate included zero, suggesting no effect of the intervention. Twelve trials used xylitol gum only as the basis of the intervention; xylitol gum significantly reduced the load of *Streptococcus mutans* (effect size − 0.46; 95% CI − 0.64 to − 0.28) in comparison to all controls. There was a moderate level of heterogeneity across the included studies. No adverse effects were recorded.

**Conclusion:**

Chewing SFG reduces the load of *Streptococcus mutans* in the oral cavity in comparison to non-chewing controls. Considering the degree of variability in the effect and the moderate quality of the trials included, there is a need for future research exploring the use SFG as a preventive measure for reducing the cariogenic oral bacterial load.

**Supplementary Information:**

The online version contains supplementary material available at 10.1186/s12903-021-01517-z.

## Background

The World Health Organisation (WHO) estimates the global burden of oral disease to affect half the world’s population. Untreated dental caries in permanent teeth affects 2.4 billion people and caries of the primary dentition affects 532 million children [1]. The financial cost of oral healthcare is high averaging 5% of total health expenditure in high income countries and oral health needs are beyond capacity in most low- and middle-income countries [1].

The presence of oral micro-organisms is an accepted associated causative factor for the development of dental caries. Preventive measures could therefore target these micro-organisms, in addition to reducing sugar consumption. One implicated micro-organism is *Streptococcus mutans* (SM) (facultatively anaerobic, gram-positive), the presence of which is associated with driving the caries process [2, 3]. The chewing of sugar-free gum (SFG) potentially provides a low cost adjunct to other caries preventive measures [4]. Its oral benefits relate to stimulating saliva, facilitating natural oral cavity clearance and delivering bacteriostatic ingredients such as xylitol and sorbitol to the oral biofilm [5, 6]. The United Kingdom (UK) Oral Health Foundation [7], the European Commission [8, 9], the European Food Safety Authority [10], and the World Dental Federation (FDI) [11], amongst other dental associations worldwide, have recognised these oral benefits.

This paper reports the findings of a systematic review of studies exploring the relationship between use of SFG and micro-organisms, specifically *Streptococcus mutans*, in the oral cavity, as part of a larger review of the role of sugar-free gum in relation to oral health. The research question addressed is, “In adults and children who chew sugar-free gum (SFG), compared with those who chew gums other than SFG (excluding sugared gum and gums with active ingredients), who do not chew gum or who use alternatives such as probiotics or fluoride varnish, what is the difference in level of oral micro-organisms, specifically *Streptococcus mutans*?”.

## Methods

### Protocol and registration

The methodology for this systematic review was registered on PROSPERO (CRD42018094676).

### Inclusion criteria

- Human participants: adults and children
- Primary research, published from 1 January 1946 to 31 August 2020
- Study designs: trials including randomised controlled trials (RCTs), crossover trials, pre-post trials, pre-post one arm trials, post-only trials and any design with a comparative arm. Crossover trials were required to have a minimum ‘washout period’ of 7 days between intervention arms.
- Full text available in English

### Exclusion criteria

- Systematic or narrative reviews.
- Non-experimental studies.
- Laboratory-based studies.
- Non-adherence to experimental allocation. That is, any trial where the original participant allocation to intervention/control had been changed on any basis, such as self-reported behaviour, assessed level of use of active intervention.
- Conference abstracts.
- Incomplete datasets.

### Interventions

Studies that had the chewing of SFG as the main intervention were included in the review. “Sugar” referred to monosaccharides (i.e. glucose, fructose, galactose) and disaccharides (i.e. sucrose, lactose, maltose) while polyols such as xylitol, sorbitol or malitol in gums satisfied the “sugar-free” criteria.

### Outcomes

The outcomes reported related to *Streptococcus mutans* specifically:

- *Streptococcus mutans* count.
- *Streptococcus mutans* trends (decline).
- *Streptococcus mutans* mean % change.

Reported adverse consequences (negative effects and harm) of SFG within the included studies, as well as acceptability and implementation methods leading to greater adherence, were collated. In addition, for each included study, data were extracted on potential effect modifiers.

### Information sources and search

**Fig. 1:**
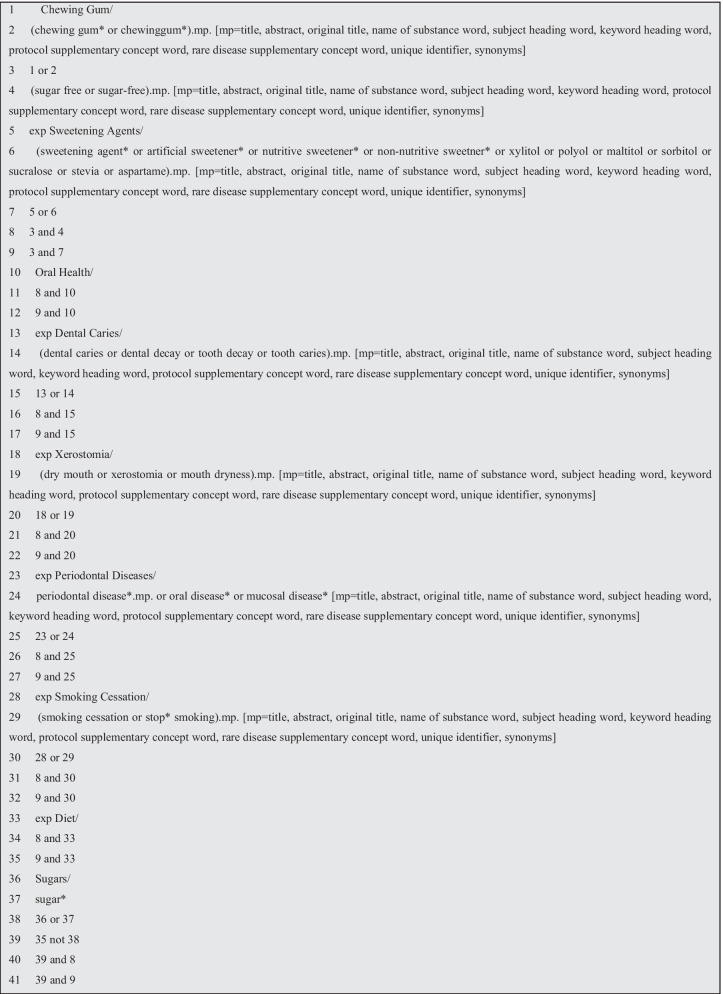
Search strategy for ovid medline, modified for other databases

An information specialist (SDG) designed and conducted the search strategy, applying it to one database (OVID Medline) initially. Both Medical Subject Headings (MESH), and free text were used as the basis for search terms with combinations of chewing gum, sugar free, caries, xerostomia, periodontal disease (see Fig. 1). The detailed search was then adapted for all the relevant databases with appropriate modifications: Ovid MEDLINE, Ovid EMBASE, Ovid PsycINFO, Scopus, Web of Science, Allied and Complimentary Medicine Database (AMED), Cochrane Central Register of Controlled Trials (CENTRAL), Open Grey, as well as searching Prospero and the Cochrane library of systematic reviews.

### Study selection

**Fig. 2:**
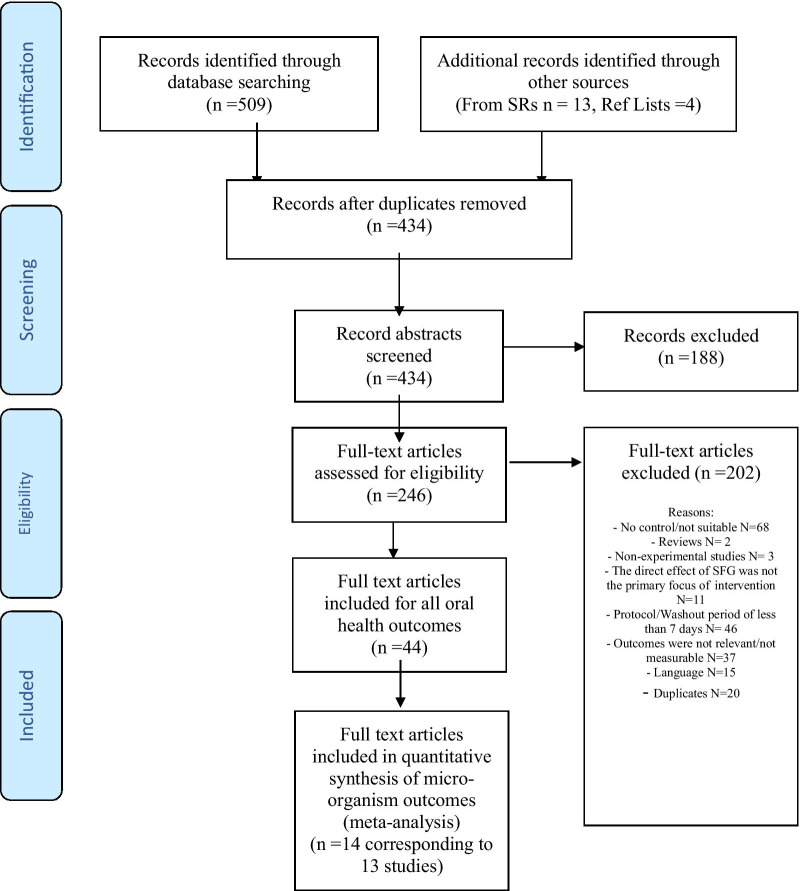
PRISMA flowchart of study identification, screening and inclusion

On the basis of the research question against the inclusion and exclusion criteria, an initial independent screening of titles and abstracts was done by two reviewers (OA/AB). All relevant full text relevant studies were checked for eligibility. Disagreements between reviewers was resolved by the input of a third reviewer (JTN). When further clarifications were required, attempts were made to contact study authors to confirm eligibility and ascertain methodological details. Following data extraction for full text review, articles were excluded if they did not meet the eligibility criteria (Fig. 2 and Additional file 1: Appendix). Fifteen papers were excluded because they were not available in English. Six of these manuscripts excluded on the basis of language related to caries outcome, whilst nine assessed plaque and salivary changes.

The references cited in the included studies were also reviewed to see if any additional papers could be identified meeting the initial inclusion criteria, resulting in seven further papers being included (JTN/MN).

Meta-analysis was undertaken using data recorded at baseline and at the end of each study. Where there was more than one publication from a single study reporting outcomes at successive time points, only data relating to the final time point were used. If more than one SFG was investigated, these results were combined and compared to the control group. An analysis of xylitol-only gums was included as it was the most frequently adopted SFG tested and the investigators wished to determine whether any recommendations could be made for xylitol SFG specifically. Where the data for either the control or SFG arms were available at both baseline and at the end of the study, the paired data were re-created using the method outlined by Borenstein et al. [12]. The correlation between the baseline and the end of study data was assumed to be 0.95 for the control and 0.65 for the SFG group. These values were selected, following a process of discussion with researchers experienced in trials of oral health outcomes. In addition, parallel analyses were performed with a near perfect correlation (0.95) with no impact on the outcomes.

### Data collection process

A pre-determined list of outcomes of interest was used to guide data extraction, developed and piloted by three investigators (OA/MN/JTN). Two investigators extracted the data from all studies, calling on the third in cases of disagreement. Twenty nine study authors were contacted, eight of whom provided additional data. Fourteen authors did not respond and a further seven responded but were unable to provide the information requested.

### Data items

Data on *Streptococcus mutans* were recorded as mentioned above. In addition, for each included study, data were extracted on the potential effect modifiers such as:

- *The intervention* who delivered it, the setting, details of gum used e.g. ingredients and concentrations, recommended usage e.g. frequency of use, duration of use.
- *Participant characteristics* age, social class, sample size, diet, pre-existing conditions, risk of population, oral hygiene details.
- *Relevant study details* number of participants in each arm at baseline and included in analysis, number of withdrawals, follow up period, washout period, unit of randomisation, unit of analysis.
- *Bibliographic details* author(s), title, journal, country of origin, year of publication, trial design.

Differences were resolved through discussion and the input of a fourth investigator if necessary (AB). With studies published across multiple manuscripts, data were extracted just once but verified across all publications to ensure optimisation of data extraction.

### Risk of bias in individual studies

Three reviewers (OA, JTN, MN) assessed all included studies independently across six domains: selection, performance, detection, attrition, reporting and ‘other’ biases using the Cochrane tool for assessing risk of bias (Modified version 1) [13]. If necessary, disagreements were resolved through discussion and with the input of a fourth investigator (AB) as required. Where a study design was not a randomised controlled trial, this was noted in the randomisation assessment in the “Risk of Bias” table (Table 2).

### Summary measures

The effect size was calculated using the procedure metaeff in Stata v15.1 (StataCorp. 2017. *Stata Statistical Software: Release 15*. College Station, TX: StataCorp LLC). The metaan command in Stata v15.1 was then used to conduct a random effects maximum likelihood meta-analysis and draw forest plots, as well as to calculate the heterogeneity between studies.

### Risk of bias across studies

Authors were contacted to clarify concerns regarding incomplete data, data in graphs or figures, pooled data, or incomplete information of key elements from the data extraction form. Missing information and/or additional clarification produced by authors was passed on to the statistician and if considered valid, these papers were included and data extraction completed.

### Changes to protocol following commencement of study

Studies with apparently incomplete outcome data were excluded unless contact with the authors could ensure that the dataset was complete. Sensitivity analyses had been planned based on the risk of bias, but were not conducted as there was little variation across the studies for this variable. In the protocol, the analytical strategy stated that analyses would include all covariates (effect modifiers), but these were not included in the analyses reported here.

## Results

A total of 14 full text articles, corresponding to 13 studies, with *Streptococcus mutans* levels as an outcome were included for analysis in this systematic review as summarised in Table 1. Figure 2 shows the PRISMA flow chart for identification of manuscripts included in this review. All 13 studies were included in the overall meta-analysis. Of these, 12 had used xylitol gum specifically as the basis of the intervention and these were considered in a separate meta-analysis. Studies lasted between 2 weeks and 18 months. Subjects were requested to chew from 2 to 5 times daily and for a length of 5–15 min. Though 6 out of the 13 trials asked participants to chew 3 times daily for 5 min.

**Table 1:** Summary characteristics of included studies

Study citation	Intervention Daily frequency/amount/chew-time		Participant characteristics	Duration of follow up	Study design	Control group	Effect size (95% CI)
Hoerman [16]	Xylitol for 10 weeks	Chewed 5 times a day for 10 min	N = 30 dental students	10 weeks	RCT	No gum	− 0.31 (− 1.21, 0.59)
Hildebrandt and Sparks [17]	Xylitol for 3 months	Chewed 3 times a day for 5 min (after each meal)	N = 151; adults	3 months	RCT	No gum	− 0.70 (− 1.09, − 0.31)
Thaweboon et al. [18]	55% xylitol and 100% xylitol for 90 days	Chewed 3 times a day for 5 min	N = 91 schoolchildren aged 10–12	90 days	Other-controlled trial	No gum	− 0.57 (− 1.00, − 0.13)
Makinen et al. [19]	Xylitol and d-glucitol for 6 months	Chewed 4 times a day for 5 min	N = 123, kindergarten children	6 months	RCT	No gum	− 0.40 (− 0.84, 0.04)
Wang et al. [20]	Sugar free gum for 14 days	Chewed 5 times a day for 10 min	N = 40 adults	2 weeks	RCT	No gum	− 0.86 (− 1.48, − 0.24)
Haresaku et al. [21]	Xylitol and malatol for 6 months	Chewed 3 times a day for 5 min (after each meal)	N = 128 adults	6 months	Patient preference non-randomised trial	No gum	− 0.10 (− 0.55, 0.34)
Calgar et al. [22]	Xylitol 21 days	Chewed 3 times a day for 10 min	N = 60 adults (age range 21–24 years)	3 weeks	RCT	Placebo gum	− 1.33 (− 2.18, − 0.48)
Campus et al. [23]	Xylitol for 9 months	Chewed 5 times a day for 5 min	N = 176 children aged 7–9	9 months	RCT	Placebo gum	− 0.01 (− 0.32, 0.31)
Hildebrandt et al. [24]	Xylitol for 3 months	Chewed 3 times a day for 5 min	N = 105 adults	3 months	RCT	No gum	− 0.76 (− 1.23, − 0.28)
Seki et al. [25]	Xylitol gum over 3 months	Chewed 4 times/day (after breakfast, lunch, snacks, and dinner) for 5 min	N = 161 children aged 3–4 years	9 months	RCT	Control gum	− 0.33 (− 0.64, − 0.02)
Alamoudi et al. [26] and Hanno et al. [27]	Xylitol gum over 3 months	Chewed 3 times a day for 5 min	N = 34 mother–child dyads	18 months	RCT	Fluoride varnish	− 0.66 (− 1.65, 0.32)
Al-Haboubi et al. [28]	Xylitol gum over 6 months	Chewed 2 times daily for 15 min	N = 186 adults aged over 60 years	6 months	RCT	No gum	− 0.23 (− 0.56, 0.10)
Ghassemi et al. [29]	Xylitol for 4 weeks	Chewed 3 times a day for 5 min	N = 50 adults (female university students)	4 weeks	RCT	Probiotic	− 0.18 (− 0.74, 0.37)

The analysis of the risk of bias within individual studies included in the review is summarised in Table 2. Of the 13 studies included in the review, 11 (84.6%) were randomised controlled trials (RCTs), one (7.7%) was a patient preference non-randomised trial and the remaining one (7.7%) has been qualified as other since the methodology is unspecified. The randomisation of participants was unclear for five (45.5%) of the RCTs and there was a high risk of bias for randomisation for one of the other study designs. Masking of participants was performed poorly for many of the trials and therefore was it was not possible to determine the level of selective reporting of outcomes in the manuscripts, which may reflect a lack of adherence with guidance on the reporting of clinical trials.

**Table 2:** Summary of risk of bias of included studies

	Study design	Randomisation	Allocation concealment	Masking of participants	Masking of outcome assessors	Incomplete outcome reporting	Selective reporting	Other bias
Hoerman [16]	RCT	Unclear	High risk	unclear	Unclear	Low risk	Low risk	Low risk
Hildebrandt and Sparks [17]	RCT	Unclear	Unclear	Unclear	Low risk	Unclear	Unclear	Unclear
Thaweboon et al. [18]	Other	Unclear [1]	Unclear	Unclear	Unclear	Unclear	Unclear	Unclear
Makinen et al. [19]	RCT	Unclear	Unclear	Low risk	Low risk	High risk	Unclear	Unclear
Wang et al. [20]	RCT	Low risk	Unclear	Unclear	Unclear	Low risk	Unclear	Unclear
Haresaku et al. [20]	Patient preference non-randomised trial	High risk [2]	Unclear	Unclear	Low risk	High risk	Unclear	Unclear
Calgar et al. [22]	RCT	Low risk	Unclear	Low risk	Low risk	Unclear	Unclear	Low risk
Campus et al. [23]	RCT	Low risk	Low risk	Low risk	Low risk	Unclear	Unclear	Low risk
Hildebrandt et al. [24]	RCT	Low risk	Unclear	High risk	Low risk	Low risk	Unclear	Unclear
Seki et al. [25]	RCT	Low risk	Low risk	Low risk	Unclear	High risk	Unclear	High risk
Alamoudi et al. [26] Hanno et al. [27]	RCT	Unclear	High risk	High risk	Unclear	Unclear	Unclear	Unclear
Al-haboubi et al. [28]	RCT	Low risk	Low risk	High risk	Low risk	Unclear	Unclear	Low risk
Ghassemi et al. [29]	RCT	Unclear	Unclear	Low risk	Unclear	Low risk	Unclear	Unclear

Participants were divided into three groups which were balanced according to their *S. mutans* counts at baseline: one control group (no supervised gum use), and two xylitol groups (supervised 55% and 100% xylitol gum use). It is unclear whether they were or not randomised after stratification

Participants’ preference for the flavour of gum was taken into account at allocation in an effort to enhance adherence to the chewing regimen

**Fig. 3:**
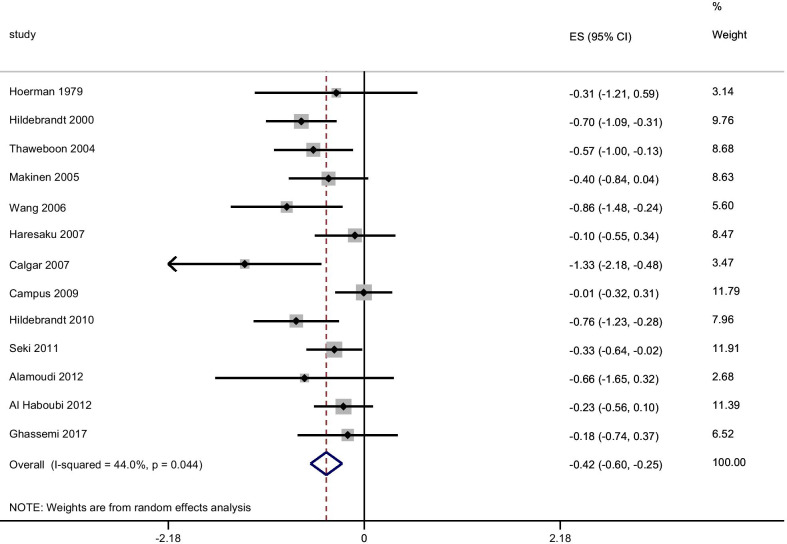
Meta-analysis of any SFG and *Streptococcus mutans* using the random-effects model by date of publication

The results of the meta-analysis are presented in Fig. 3. The use of SFG significantly reduced the *Streptococcus mutans* load (effect size − 0.42 (95% CI − 0.60 to − 0.25) in comparison to all controls. There was moderate heterogeneity between studies (I^2^ = 44%). Changing the correlation between the baseline and end of study data to 0.95 for the SFG group gave an effect size of − 0.55 (95% CI − 0.80 to − 0.30) and I^2^ = 72.7%. In seven of the 13 studies, the confidence intervals of the effect size estimate included zero, suggesting no effect of the intervention.

In the second meta-analysis of trials involving xylitol gum only, this gum significantly reduced *Streptococcus mutans* in comparison to all controls (effect size − 0.46; 95% CI − 0.64 to − 0.28). Again, there was a moderate level of heterogeneity between studies with I^2^ = 42.8%. No adverse events were reported in any of the studies.

A sensitivity analysis was undertaken including only studies with adult participants. This made no significant difference to the effect sizes noted.

## Discussion

This systematic review and meta-analysis confirm the *Streptococcus mutans*-reducing effect of xylitol-containing SFG and, as such, the potential for SFG to be considered as an adjunct to preventive oral health care regimes for dental caries management. Eleven of the 13 studies examined xylitol only. Xylitol competes with mono- and polysaccharides in the metabolic pathway but cannot be actively metabolised by *Streptococcus mutans* and therefore does not produce a decrease in salivary pH from lactic acid production. Xylitol also inhibits the attachment of *Streptococcus mutans* to the tooth surface [14]. The effect of chewing xylitol-containing SFG on *Streptococcus mutans* counts is potentially long-lasting, with evidence suggesting that it lowers the oral bacterial load for up to five years, after 2 years of use [15].

The main outcome analysed in these studies was the effect of SFG on levels of *Streptococcus mutans* with ten of the studies looking at the *Streptococcus mutans* count, one looking at average percentage change [20] and others at *Streptococcus mutans* decline [26, 30]. The overall effect size for all sugar-free gums (− 0.42) and for xylitol-containing gums (− 0.46) compares favourably to other preventive interventions including oral health education [31] and supervised toothbrushing programmes alone [32]. No adverse events were reported but this may be related to absence of evidence, as few studies reported active attempts to gather data on possible adverse events.

The search strategy was wide ranging and comprehensive, including the review of the citations in all studies identified in the electronic searches. A strength of the studies reviewed is that the majority (all bar one) were randomised control trials, eight studies reporting on adults and five on children, with sample sizes of 30 participants minimum and duration of follow up from four weeks to 18 months, which fits into accepted review/recall protocols for caries management. It is important to acknowledge that the level of *Streptococcus mutans* is only one component of an individual’s risk/susceptibility of dental caries. The clinical significance of the changes in levels of *Streptococcus mutans* identified in this study are yet to be determined, particularly on an individual basis where there are other risk factors present. However, the beneficial impact of chewing SFG on caries has been demonstrated and may be in part mediated through changes in levels of *Streptococcus* mutans [33]. As part of a preventive strategy, SFG has the advantage of being readily accessible. In addition, the use of other xylitol-containing products is increasing, particularly in the dental setting with products such as toothpaste, rinses, and sprays including xylitol in their formulations.

*Streptococcus mutans* has been linked to certain cardiovascular pathologies as it is the most prevalent bacterial species detected in extirpated heart valve tissues (68.6%), as well as in atheromatous plaques (74.1%) [34]. *Streptococcus sanguinis*, closely related to *Streptococcus mutans*, has been linked to infective endocarditis [35]. Xylitol studies in rats have shown to increase collagen production in the skin, increase bone volume and bone mineral content and improve digestive health. These findings all need further investigation and in particular, more studies in humans [36–42]. Xylitol also does not appear to affect blood glucose and insulin levels [43–45]. Xylitol may also have antimicrobial effect on *Candida albicans* and can be used as an effective element in gums, toothpastes, and antimicrobial mouthwashes, especially in patients with candidiasis [46]. Therefore, the *Streptococcus mutans*-reducing effect of xylitol-containing SFG is not only of value from an oral health perspective but also potentially for wider general health benefits. The recommendation would be to further research the effect of xylitol use on *Streptococcus mutans* counts in relation to the above-mentioned general health-related outcomes. Any health benefits of using xylitol products must be considered in relation to the reported role of xylitol in gut dysbiosis and metabolic acidosis [45].

There was a high degree of heterogeneity among the studies in terms of the length of time, and the dosage of xylitol gum which formed the basis of the intervention, as well as the during of follow-up. Interestingly, the study which found little difference between the chewing group and the control (Campus 2009) had a long-expected duration of chewing (9 months) coupled with an equally long follow-up period (9 months). The lack of significant difference may be the result of the challenge of maintaining compliance with the intervention in children over this period. Studies with shorter intervention periods and shorter follow ups such as Wang et al. and Caglar et al., show large effect sizes and may reflect greater participant adherence. There are implications for the planning of interventions based on chewing gum—ensuring long term adherence may require specific support.

It is important to take into consideration the limitations of the current review. The search terms did not specifically include *Streptococcus mutans*. This was the result of the broad aim of the overarching registered systematic review protocol. This sought to focus on a range of oral health outcomes including levels of micro-organisms. It is possible that the exclusion of this specific search term may have meant that some relevant articles were not identified. The exclusion of articles not written in English may have led to bias. Hand-searching of relevant journals was not undertaken, but the authors did undertake a review of the references cited in the manuscripts identified for review, to the point where no new manuscripts were identified. There was a moderate level of heterogeneity in the trials in terms of the dosage and frequency of use of the SFGs, as well as in the length of follow-up, which makes it more difficult to draw conclusions concerning the ideal regime for the use of SFGs. However, the findings do suggest that regardless of variation in the manner of their use, there is a potential for reduction in *Streptococcus mutans* levels. Further research could focus on ascertaining the optimal duration and pattern of use of SFG; and, might explore the pharmacodynamics of the impact of xylitol on levels of *Streptococcus mutans*. From the present studies it is unclear the time point at which the maximal impact is reached. Further research might explore the pharmacodynamics of the impact of xylitol on levels of *Streptococcus mutans*.

The possibility of publication bias was not explored and there were insufficient data to conduct a sensitivity analysis to identify the variables contributing to the heterogeneity. From an analysis of the risk of bias, the quality of evidence was variable and there is a clear need for better designed trials which measures of participant adherence to the intervention and include reporting of adverse events, or their absence. Lastly, levels of *Streptococcus mutans* as measured in the studies included in this study are only one measure of the activity of the micro-organism in the oral cavity. Future research could seek to systematically review and synthesis the published literature appraising the impact of chewing SFGs on the qualitative determination of *Streptococcus mutans* activity, particularly amongst communities where caries risk is higher.

## Conclusion

In conclusion there is evidence to support the use of sugar-free gum in the control of *Streptococcus mutans* counts, which in turn relate directly to caries progression in children and adults. Further research should be undertaken to assess the use SFG for the delivery of xylitol as a justifiable and achievable preventative measure in Dental Public Health.

## Supplementary Information

**Additional file 1**. Prisma Flow chart detail of the exclusions at full text review.

## Data Availability

All the data used in the systematic review presented in this publication is available upon request.

## Abbreviations

MN: Melanie Nasseripour
JTN: Jonathon Timothy Newton
OA: Oluwatunmise Awojobi
FW: Fiona Warburton
SDG: Sonya Di Giorgio
JEG: Jennifer Elizabeth Gallagher
AB: Avijit Banerjee
SFG: Sugar-free gum
WHO: World Health Organisation
UK: United Kingdom
EFSA: European Food Safety Authority
FDI: Fédération Dentaire Internationale
ES: Standardised effect size
CI: Confidence interval
RCT: Randomised control trial
MESH: Medical Subject Headings
Ovid: Object, view and interaction design
EMBASE: Excerpta Medica DataBASE
MEDLINE: Medical literature analysis and retrieval system online
PsycINFO: Psychological information database
AMED: Allied and Complimentary Medicine Database
CENTRAL: Cochrane Central Register of Controlled Trials
PICO: Population intervention comparison outcome
PF: Prevention factor
SMD: Standardised mean difference
