# Supplementary material for: A systematic review and meta-analysis of the role of sugar-free chewing gum on Streptococcus mutans
Source: BMC Oral Health. 2021 Apr 29;21:217. doi: 10.1186/s12903-021-01517-z (PMC8082871; doi:10.1186/s12903-021-01517-z)
Supplement: Supplementary file 1 — Additional file 1. Prisma Flow chart detail of the exclusions at full text review. [file 12903_2021_1517_MOESM1_ESM.docx]

Prisma Flow chart detail of the exclusions at full text review

Reasons:

- No control/not suitable N=67

1. Milgrom, P., et al. (2006). "Mutans streptococci dose response to xylitol chewing gum." Journal of Dental Research 85(2): 177-181.
2. Isotupa, K. P., et al. (1995). "Effect of polyol gums on dental plaque in orthodontic patients." American Journal of Orthodontics & Dentofacial Orthopedics 107(5): 497-504.
3. Wennerholm, K., et al. (1994). "Effect of xylitol and sorbitol in chewing-gums on mutans streptococci, plaque pH and mineral loss of enamel." Caries Research 28(1): 48-54.
4. Cocco, F., et al. (2017). "The caries preventive effect of 1-year use of low-dose xylitol chewing gum. A randomized placebo-controlled clinical trial in high-caries-risk adults." Clinical Oral Investigations 21(9): 2733-2740.
5. Iijima, Y., et al. (2004). "Acid resistance of enamel subsurface lesions remineralized by a sugar-free chewing gum containing casein phosphopeptide-amorphous calcium phosphate." Caries Research 38(6): 551-556.
6. Fraga, C. P., et al. (2010). "Use of chewing gum containing 15% of xylitol and reduction in mutans streptococci salivary levels." Pesquisa Odontologica Brasileira = Brazilian Oral Research 24(2): 142-146.
7. Subramaniam, P. and P. Suresh Babu (2011). "Effect of polyol gums on salivary S mutans levels." Journal of Clinical Pediatric Dentistry 36(2): 145-147.
8. Cai, F., et al. (2009). "Remineralization of enamel subsurface lesions by chewing gum with added calcium." Journal of Dentistry 37(10): 763-768.
9. Campus, G., et al. (2011). "Effect of a sugar-free chewing gum containing magnolia bark extract on different variables related to caries and gingivitis: A randomized controlled intervention trial." Caries Research 45(4): 393-399.
10. Manton, D. J., et al. (2008). "Remineralization of enamel subsurface lesions in situ by the use of three commercially available sugar-free gums." International Journal of Paediatric Dentistry 18(4): 284-290.
11. Simons, D., et al. (1997). "The effect of chlorhexidine/xylitol chewing-gum on cariogenic salivary microflora: a clinical trial in elderly patients." Caries Research 31(2): 91-96.
12. Simons, D., et al. (1999). "The effect of xylitol and chlorhexidine acetate/xylitol chewing gums on plaque accumulation and gingival inflammation." Journal of clinical periodontology 26(6): 388-391.
13. Wessel, S. W., et al. (2017). "Self-perceived mouthfeel and physico-chemical surface effects after chewing gums containing sorbitol and Magnolia bark extract." European journal of oral sciences 125(5): 379-384.
14. Morgan, M. V., et al. (2008). "The anticariogenic effect of sugar-free gum containing CPP-ACP nanocomplexes on approximal caries determined using digital bitewing radiography." Caries Research 42(3): 171-184.
15. Suyama, E., et al. (2011). "Remineralization and acid resistance of enamel lesions after chewing gum containing fluoride extracted from green tea." Australian dental journal 56(4): 394-400.
16. Soderling, E., et al. (2015). "Effects of short-term xylitol gum chewing on the oral microbiome." Clinical Oral Investigations 19(2): 237-244.
17. BOTS CP, BRAND HS, VEERMAN ECI, VAN AMERONGEN BM,NIEUW AMERONGEN AV. Preferences and saliva stimulation of eight different chewing gums. Int Dent J 2004; 54: 143–148. From WESSEL
18. Campus, G., et al. (2013). "Six months of high-dose xylitol in high-risk caries subjects--a 2-year randomised, clinical trial." Clinical Oral Investigations 17(3): 785-791. - ??Same study as Caries Research 43(6): 455-461.
19. Kaae, J. K., et al. (2016). "Xerostomia after radiotherapy for oral and oropharyngeal cancer: Increasing salivary flow with tasteless sugar-free chewing gum." Frontiers in Oncology 6.
20. Cronin, M., et al. (1994). "Three clinical trials comparing xylitol- and sorbitol-containing chewing gums for their effect on supragingival plaque accumulation." Journal of Clinical Dentistry 5(4): 106-109.

# [Wennerholm K](https://www.ncbi.nlm.nih.gov/pubmed/?term=Wennerholm%20K%5BAuthor%5D&cauthor=true&cauthor_uid=2740837)^1^, [Emilson CG](https://www.ncbi.nlm.nih.gov/pubmed/?term=Emilson%20CG%5BAuthor%5D&cauthor=true&cauthor_uid=2740837)., 1989. Effect of sorbitol- and xylitol-containing chewing gum on salivary microflora, saliva, and oral sugar clearance. [Scand J Dent Res.](https://www.ncbi.nlm.nih.gov/pubmed/2740837) 1989 Jun;97(3):257-62.??

1. Martinez-Pabon, M. C., et al. (2014). "Comparison of the effect of two sugar-substituted chewing gums on different caries- and gingivitis-related variables: a double-blind, randomized, controlled clinical trial." Clinical Oral Investigations 18(2): 589-598.
2. Dawes, C. and Dong, C., 1995. The flow rate and electrolyte composition of whole saliva elicited by the use of sucrose-containing and sugar-free chewing-gums. Archives of oral biology, 40(8), pp.699-705. (From: Cai, 2009)
3. Pizzo, G., et al. (2007). "The effects of sugar-free chewing gums on dental plaque regrowth: A comparative study." Journal of Dentistry 35(6): 503-508.
4. Holgerson, P. L., et al. (2007). "Dental plaque formation and salivary mutans streptococci in schoolchildren after use of xylitol-containing chewing gum." International Journal of Paediatric Dentistry 17(2): 79-85.
5. Kumar, S., et al. (2013). "Comparative evaluation of the effects of xylitol and sugar-free chewing gums on salivary and dental plaque pH in children." Journal of Indian Society of Pedodontics and Preventive Dentistry 31(4): 240-244.
6. Emamieh, S., et al. (2015). "The effect of two types chewing gum containing casein phosphopeptide-amorphous calcium phosphate and xylitol on salivary Streptococcus mutans." Journal of conservative dentistry : JCD 18(3): 192-195.
7. Kimbrough, C., Chun, M., Dela Roca, G., & Lau, B. H. S. (2002). Pycnogenol® chewing gum minimizes gingival bleeding and plaque formation. Phytomedicine, 9(5), 410-413.
8. Tulsani, S. G., et al. (2014). "The effect of Propolis and Xylitol chewing gums on salivary Streptococcus mutans count: a clinical trial." Indian Journal of Dental Research 25(6): 737-741.
9. Vogel, G.L., Zhang, Z., Carey, C.M., Ly, A., Chow, L.C. and Proskin, H.M., (1998). Composition of plaque and saliva following a sucrose challenge and use of an a-tricalcium-phosphate-containing chewing gum. Journal of dental research, 77(3), pp.518-524.
10. Scheie, A. A., et al. (1998). "The effects of xylitol-containing chewing gums on dental plaque and acidogenic potential." Journal of Dental Research 77(7): 1547-1552. .
11. Soderling, E., et al. (1976). "Turku sugar studies XXI. Xylitol, sorbitol-, fructose- and sucrose-induced physico-chemical changes in saliva." Acta odontologica Scandinavica 34(6): 397-403.
12. Gazi, M. I. (1991). The finding of antiplaque features in Acacia Arabica type of chewing gum. Journal of clinical periodontology, 18(1), 75-77.
13. Porciani, P. F. and S. Grandini (2012). "The effect of zinc acetate and magnolia bark extract added to chewing gum on volatile sulfur-containing compounds in the oral cavity." The Journal of clinical dentistry 23(3): 76-79.
14. Scheinin, A., et al. (1975). "Turku sugar studies XVIII. Incidence of dental caries in relation to 1-year consumption of xylitol chewing gum." Acta odontologica Scandinavica 33(5): 269-278.
15. Larmas, M., et al. (1976). "Turku sugar studies XX. Microbiological findings and plaque index values in relation to 1-year use of xylitol chewing gum." Acta odontologica Scandinavica 34(6): 381-396.
16. Assev, S. and G. Rolla (1994). "Does the presence of xylitol in a sorbitol-containing chewing gum affect the adaptation to sorbitol by dental plaque?" Scandinavian Journal of Dental Research 102(5): 281-283
17. Vogel, G. L., et al. (2000). "Composition of plaque and saliva following use of an alpha-tricalcium-phosphate-containing chewing gum and a subsequent sucrose challenge." Journal of Dental Research 79(1): 58-62.
18. Shinga-Ishihara, C., et al. (2012). "Xylitol carryover effects on salivary mutans streptococci after 13 months of chewing xylitol gum." Caries Research 46(6): 519-522.
19. Milgrom P, Ly KA, Roberts MC et al. Mutans streptococci dose response to xylitol chewing gum. J Dent Res 2006 85: 177-181. From Makinen
20. Ainamo, J. and H. Etemadzadeh (1987). "Prevention of plaque growth with chewing gum containing chlorhexidine acetate." Journal of clinical periodontology 14(9): 524-527.
21. Eghbali, A., et al. (2016). "Effect of Chewing Gum on Oral Mucositis in Children Undergoing Chemotherapy: A Randomized Controlled Study." Iranian Journal of Pediatric Hematology and Oncology 6(1): 9-14.
22. Lee, E. J., et al. (2009). "Preventive effect of sugar-free chewing gum containing maltitol on dental caries in situ." Food Science and Biotechnology 18(2): 432-435.
23. Möller, I. J. and S. Poulson (1973). "The effect of sorbitol‐containing chewing gum on the incidence of dental caries; plaque and gingivitis in Danish schoolchildren." Community Dentistry and Oral Epidemiology 1(2): 58-67.
24. Petersen, P. E. and N. Razanamihaja (1999). "Carbamide-containing polyol chewing gum and prevention of dental caries in schoolchildren in Madagascar." International Dental Journal 49(4): 226-230.
25. Makinen, K. K., et al. (1995). "Saliva stimulants and the oral health of geriatric patients." Advances in Dental Research 9(2): 125-126.
26. Rosing, C. K., et al. (2009). "Effect of chewing gums on the production of volatile sulfur compounds (VSC) in vivo." Acta Odontologica Latinoamericana 22(1): 11-14.
27. Splieth, C. H., et al. (2009). "Effect of xylitol and sorbitol on plaque acidogenesis." Quintessence International 40(4): 279-285.
28. Kandelman, D., et al. (1988). "Collaborative WHO xylitol field study in French Polynesia. I. Baseline prevalence and 32-month caries increment." Caries Research 22(1): 55-62.
29. Smith AJ, Moran J, Dangler LV, Leight RS, Addy M. 1996 The efficacy of an anti-gingivitis chewing gum. J Clin Periodontol; 23: 19–23.
30. Lin, Y. T. and R. E. Corpron (1991). "In vivo study of fluoride chewing gum for the remineralization of human root lesions." Changgeng Yi Xue Za Zhi 14(3): 174-185.
31. Topcuoglu, N., et al. (2015). "Antibacterial Effect of Kenger Gum on Mutans Streptococci and Its Cytotoxic Effect on the 3T3 Fibroblast Cell Line." Oral Health & Preventive Dentistry 13(2): 157-162.
32. Smith, C. A., et al. (2004). "The effect of chewing urea-containing gum on plaque acidogenic and alkaligenic parameters." Caries Research 38(2): 124-129.
33. Sjogren, K., et al. (2002). "Fluoride and urea chewing gums in an intra-oral experimental caries model." Caries Research 36(1): 64-69.
34. Keller, M. K., Bardow, A., Jensdottir, T., Lykkeaa, J., & Twetman, S. (2012). Effect of chewing gums containing the probiotic bacterium Lactobacillus reuteri on oral malodour. Acta Odontologica Scandinavica, 70(3), 246-250.
35. Kolahi, J., Soolari, A., Ghalayani, P., Varshosaz, J., & Fazilaty, M. (2008). Newly formulated chlorhexidine gluconate chewing gum that gives both anti-plaque effectiveness and an acceptable taste: a double blind, randomized, placebo-controlled trial. Journal of the International Academy of Periodontology, 10(2), 38-44.
36. Masalin, K. (1992). "Caries-risk-reducing effects of xylitol-containing chewing gum and tablets in confectionery workers in Finland." Community Dental Health 9(1): 3-10.
37. Cagetti, M. G., et al. (2020). "Long-term efficacy of Magnolia Bark Extract and Xylitol administered through chewing gums on caries in adults: A 2-year randomized controlled intervention trial." Journal of Functional Foods68: 9.
38. Kaur, K., et al. (2018). "Effect of chewing gums containing probiotics and xylitol on oral health in children: A randomized controlled trial." Journal of International Oral Health10(5): 237-243.
39. Oza, S., et al. (2018). "To Determine the Effect of Chewing Gum Containing Xylitol and Sorbitol on Mutans Streptococci and Lactobacilli Count in Saliva, Plaque, and Gingival Health and to Compare the Efficacy of Chewing Gums." Journal of International Society of Preventive and Community Dentistry8(4): 354-360.
40. Padminee, K., et al. (2018). "Effectiveness of casein phosphopeptide-amorphous calcium phosphate and xylitol chewing gums on salivary pH, buffer capacity, and Streptococcus mutans levels: An interventional study." Indian journal of dental research : official publication of Indian Society for Dental Research29(5): 616-621.
41. Shinde, M. R. and J. Winnier (2020). "Comparative evaluation of Stevia and Xylitol chewing gum on salivary Streptococcus mutans count - A pilot study." Journal of clinical and experimental dentistry12(6): e568-e573.
42. Fernando, J. R., et al. (2019). "The prebiotic effect of CPP-ACP sugar-free chewing gum." Journal of Dentistry91: 103225.
43. Gao, Q., et al. (2018). "The Efficacy of a Chewing Gum Containing Phyllanthus emblica Fruit Extract in Improving Oral Health." Current Microbiology75(5): 604-610.
44. Nct (2018). "Xylitol and Sorbitol Effects on the Oral Microbiome." <https://clinicaltrials.gov/show/NCT03668015>.
45. Nct (2019). "Impact of Chewing Gum With CPP-ACP on Tooth Mineralisation in Situ." <https://clinicaltrials.gov/show/NCT03971084>.
46. Rafeek, R., et al. (2019). "Xylitol and sorbitol effects on the microbiome of saliva and plaque." Journal of Oral Microbiology11(1): 1536181.
47. Watanabe, K., et al. (2018). CHECK WHAT IS IN PLACEBO GUM "Effects of French Pine Bark Extract Chewing Gum on Oral Malodor and Salivary Bacteria." Journal of Nutritional Science and Vitaminology64(3): 185-191.

- Reviews N= 2

1. Tsao, C. E. and M. V. Morgan (2005). "Does chewing sucrose-free chewing gum after meals reduce the development of carious lesions?" Medical Journal of Australia 182(2): 85-86.
2. Gray, D. L. (2012). "The wichita xylitol project-a viable in-school alternative to fluoride for caries prevention." Fluoride 45(4): 323-328.

- Non-experimental studies N= 3

1. Soderling, E., et al. (1991). "Long-term xylitol consumption and mutans streptococci in plaque and saliva." Caries Research 25(2): 153-157.
2. Isogangas, P., et al. (1993). "Long-term effect of xylitol chewing gum in the prevention of dental caries: a follow-up 5 years after termination of a prevention program." Caries Research 27(6): 495-498.
3. Isokangas, P., et al. (1991). "Dental caries and mutans streptococci in the proximal areas of molars affected by the habitual use of xylitol chewing gum." Caries Research 25(6): 444-448.

- Laboratory-based studies N=0
- Non-adherence to allocation N= 0
- Conference abstracts N=0

- The direct effect of SFG was not the primary focus of intervention N=11

1. Frost, P. M., et al. (2002). "Patient preferences in a preliminary study comparing an intra-oral lubricating device with the usual dry mouth lubricating methods." British Dental Journal 193(7): 403-408. –
2. Bots, C. P., et al. (2005). "The management of xerostomia in patients on haemodialysis: comparison of artificial saliva and chewing gum." Palliative Medicine 19(3): 202-207.
3. Kargul, B., et al. (1998). "Inhaler medicament effects on saliva and plaque pH in asthmatic children." Journal of Clinical Pediatric Dentistry 22(2): 137-140.
4. Greenstein, R. B. N., Goldberg, S., Marku-Cohen, S., Sterer, N., & Rosenberg, M. (1997). Reduction of oral malodor by oxidizing lozenges. Journal of periodontology, 68(12), 1176-1181.
5. Simons D, Baker P, Knott D et al. Attitudes of carers and the elderly occupants of residential homes to antimicrobial chewing gum as an aid to oral health. Br Dent J 1999;187:612–615.
6. Machiulskiene, V., et al. (2002). "Determinants of dropout in a community intervention trial on the caries-preventive effect of chewing gums." Journal of Public Health Dentistry 62(1): 21-27.
7. Autio, J. T. and F. J. Courts (2001). "Acceptance of the xylitol chewing gum regimen by preschool children and teachers in a Head Start program: a pilot study." Pediatric Dentistry 23(1): 71-74.
8. Dodds, M. W. J., et al. (1991). "The effect of increased mastication by daily gum-chewing on salivary-gland output and dental plaque acidogenicity." Journal of Dental Research 70(12): 1474-1478.
9. Dodds, M. W. J. and D. A. Johnson (1993). "Influence of mastication on saliva, plaque pH and masseter muscle activity in man." Archives of Oral Biology 38(7): 623-626.
10. Makinen, K. K., et al. (1998). "Physical, chemical, and histologic changes in dentin caries lesions of primary teeth induced by regular use of polyol chewing gums." Acta odontologica Scandinavica 56(3): 148-156.
11. Alamoudi, N. M., et al. (2012). "Impact of maternal xylitol consumption on mutans streptococci, plaque and caries levels in children." Journal of Clinical Pediatric Dentistry 37(2): 163-166.

- Protocol/Washout period of less than 7 days N= 45+1=46

1. Mouton, C., et al. (1975). "Effect on plaque of a xylitol-containing chewing-gum: A clinical and biochemical study." Acta odontologica Scandinavica 33(1): 33-40.
2. Assev, S. and G. Rolla (1995). "Effect of xylitol-containing chewing gum on sorbitol metabolism in dental plaque." European journal of oral sciences 103(2 ( Pt 1)): 103-105.
3. Assev, S., et al. (1996). "Xylitol fermentation by human dental plaque." European journal of oral sciences 104(4 (Pt 1)): 359-362.
4. Gopinath, V. K., et al. (1997). "The effect of chewing gums on acidogenicity of plaque after a sucrose challenge." The Journal of clinical pediatric dentistry 22(1): 77-81.
5. Park, K. K., et al. (1990). "The impact of chewing sugarless gum on the acidogenicity of fast-food meals." American Journal of Dentistry 3(6): 231-235.
6. Park, K. K., et al. (1993). "Effect of time and duration of sorbitol gum chewing on plaque acidogenicity." Pediatric Dentistry 15(3): 197-202.
7. Dawes C, Macpherson LMD. Effects of nine different chewing gums and lozenges on salivary flow rate and pH. Caries Res 1992;26:176–182.
8. Mouton, C., et al. (1975). "Effect on plaque of a xylitol-containing chewing-gum: A pilot study." Acta odontologica Scandinavica 33(1): 27-31.
9. Jensen, M. E. (1986). "Effects of chewing sorbitol gum and paraffin on human interproximal plaque pH." Caries Research 20(6): 503-509.
10. Davies, A. N. (2000). "A comparison of artificial saliva and chewing gum in the management of xerostomia in patients with advanced cancer." Palliative Medicine 14(3): 197-203.
11. Ainamo, J., et al. (1977). "Growth of plaque while chewing sucrose and sorbitol flavoured gum." Journal of clinical periodontology 4(3): 151-160.
12. Rios, D., et al. (2006). "Effect of salivary stimulation on erosion of human and bovine enamel subjected or not to subsequent abrasion: An in situ/ex vivo study." Caries Research 40(3): 218-223.
13. Etemadzadeh, H. (1991). "Plaque-growth inhibiting effect of chewing gum containing urea hydrogen peroxide." Journal of clinical periodontology 18(5): 337-340.
14. Imfeld, T., et al. (1995). "Effect of urea in sugar-free chewing gums on ph recovery in human dental plaque evaluated with 3 different methods." Caries Research 29(3): 172-180.
15. Antunes, D. P., et al. (2015). "Buffer capacity of saliva as a function of time after consumption of sugary, sugar-free and probiotic chewing gums." Pesquisa Brasileira em Odontopediatria e Clinica Integrada 15(1): 153-161.
16. Ballal, R. K., et al. (2016). "Effect of Chewing Bicarbonate-containing Sugar-free Gum on the Salivary pH: An in vivo Study." International journal of clinical pediatric dentistry 9(1): 35-38.
17. Hanham, A. and M. Addy (2001). "The effect of chewing sugar-free gum on plaque regrowth at smooth and occlusal surfaces." Journal of clinical periodontology 28(3): 255-257.
18. Olsson, H., et al. (1991). "The effect of a chewing gum on salivary secretion, oral mucosal friction, and the feeling of dry mouth in xerostomic patients." Acta odontologica Scandinavica 49(5): 273-279.
19. Pluss, E. M. (1978). "Effect on plaque growth of xylitol and sucrose-containing chewing gums." Journal of clinical periodontology 5(1): 35-40.
20. Murtomaa, H., et al. (1993). "The use of Xylitol chewing gum in oral health promotion for Finnish students." Health Promotion International 8(4): 271-274.
21. Anderson, L. A. and R. Orchardson (2003). "The effect of chewing bicarbonate-containing gum on salivary flow rate and pH in humans." Archives of Oral Biology 48(3): 201-204.
22. Lee, I. K. and C. F. Schachtele (1992). "Effect of gum chewing following food ingestion on the pH of interproximal dental plaque." Quintessence International 23(7): 455-459.
23. Manning, R. H., et al. (1992). "Effects of chewing gums sweetened with sorbitol or a sorbitol/xylitol mixture on the remineralisation of human enamel lesions in situ." Caries Research 26(2): 104-109.
24. Vantipalli, U. K., et al. (2017). "Effect of three commercially available chewing gums on salivary flow rate and pH in cariesactive and cariesfree children: An in vivo study." Journal of Indian Society of Pedodontics and Preventive Dentistry 35(3): 254-259.
25. Topitsoglou, V., et al. (1983). "Effect of chewing gums containing xylitol, sorbitol or a mixture of xylitol and sorbitol on plaque formation, pH changes and acid production in human dental plaque." Caries Research 17(4): 369-378.
26. Paice, E. M., et al. (2011). "The erosive effects of saliva following chewing gum on enamel and dentine: an ex vivo study." British Dental Journal 210(3): E3
27. Koparal, E., et al. (2000). "Effect of chewing gum on plaque acidogenicity." Journal of Clinical Pediatric Dentistry 24(2): 129-132.
28. Park, K. K., et al. (1990). "Effect of sorbitol gum chewing on plaque pH response after ingesting snacks containing predominantly sucrose or starch." American Journal of Dentistry 3(5): 185-191.
29. Silwood CJL, Grootveld MC, Lynch E: A multifactorial investigation of the ability of oral health care products (OHCPs) to alleviate oral malodour(2001) J Clin Periodontol ; 28: 634–641. C Munksgaard, 2001.
30. Sato S, Yoshinuma N, Ito K et al (1998) The inhibitory effect of funoran and eucalyptus extract-containing chewing gum on plaque formation. J Oral Sci 1998; 40: 115–117.
31. Lin, Y. T., et al. (2001). "Effects of fluoride chewing gum on stimulated salivary flow rate and fluoride content." Chang Gung Medical Journal 24(1): 44-49.
32. Fure, S., et al. (1998). "Effect of three months' frequent use of sugar-free chewing gum with and without urea on calculus formation." Journal of Dental Research 77(8): 1630-1637.
33. Grenby, T. H., et al. (1983). "A clinical trial to compare the effects of xylitol and sucrose chewing-gums on dental plaque growth." Dental Health 22(3): 11-14, 16.
34. Igarashi, K., et al. (1988). "Effect of chewing gum containing sodium bicarbonate on human interproximal plaque pH." Journal of Dental Research 67(3): 531-535.
35. Jensen, M. E. (1988). "Effects of chewing sorbitol gum on human salivary and interproximal plaque pH." Journal of Clinical Dentistry 1(1): 6-19.
36. Jensen, M. E. and J. S. Wefel (1989). "Human plaque pH responses to meals and the effects of chewing gum." British Dental Journal 167(6): 204-208.
37. Mouton, C. (1983). "The efficacy of gum chewing and xylitol to reduce oral glucose clearance time." Journal (Canadian Dental Association) 49(9): 655-660.
38. Rekola, M. (1988). "Acid production from xylitol products in vivo and in vitro." Proceedings of the Finnish Dental Society 84(1): 39-44.
39. Shannon, I. L. and W. J. Frome (1973). "Enhancement of salivary flow rate and buffering capacity." Journal of the Canadian Dental Association 39(3): 177-181.
40. Vratsanos, S. M. and I. D. Mandel (1981). "The effect of sucrose and hexitol--containing chewing gums on plaque acidogenesis in vivo." Pharmacology & Therapeutics in Dentistry 6(3-4): 87-91.
41. Park, K. K., et al. (1995). "Effect of chewing gums on plaque pH after a sucrose challenge." Journal of Dentistry for Children 62(3): 180-186.
42. Markovic, N., et al. (1988). "Sorbitol gum in xerostomics: the effects on dental plaque pH and salivary flow rates." Gerodontology 7(2): 71-75.
43. Abelson, D. C., et al. (1990). "The effect of chewing sorbitol-sweetened gum on salivary flow and cemental plaque pH in subjects with low salivary flow." Journal of Clinical Dentistry 2(1): 3-5.
44. Lif Holgerson, P., et al. (2006). "Xylitol concentration in saliva and dental plaque after use of various xylitol-containing products." Caries Research 40(5): 393-397.
45. Addy M, Perriam E, Sterry A: Effects of sugared and sugar free chewing gum on the accumulation of plaque and debris on teeth. J ClinPeriodontol1982;9:346–354.
46. Gul, P., et al. (2018). "Anticariogenic potential of white cheese, xylitol chewing gum, and black tea." European journal of dentistry12(2): 199-203.

- Outcomes were not relevant/not measurable N=30+7=37

1. Thaweboon, S., et al. (2009). "Remineralization of enamel subsurface lesions by xylitol chewing gum containing funoran and calcium hydrogenphosphate." Southeast Asian Journal of Tropical Medicine & Public Health 40(2): 345-353.
2. Yévenes, I., et al. (2014). "Effects of chlorophenol / hydrogen peroxide versus xylitol or chlorhexidine as chewing gum on salivary flow rate, pH, buffer capacity and salivary Streptococcus mutans scores." Revista Odonto Ciencia 29(3): 81-86.
3. Suda, R., et al. (2006). "The effect of adding calcium lactate to xylitol chewing gum on remineralization of enamel lesions." Caries Research 40(1): 43-46.
4. Gueimonde, L., et al. (2016). "Supplementation of xylitol-containing chewing gum with probiotics: a double blind, randomised pilot study focusing on saliva flow and saliva properties." Food & Function 7(3): 1601-1609.
5. Merikallio, M. C. and E. Soderling (1995). "Xylitol as a plaque-control agent in military conditions." Military Medicine 160(5): 256-258.
6. Paula, V. A., et al. (2010). "Antimicrobial effects of the combination of chlorhexidine and xylitol." British Dental Journal 209(12): E19.
7. Komarov, G. N., et al. (2017). "Dental plaque regrowth studies to evaluate chewing gum formulations incorporating magnolia bark extract." Journal of Functional Foods 37: 612-617.
8. Autio, J. T. (2002). "Effect of xylitol chewing gum on salivary Streptococcus mutans in preschool children." Journal of Dentistry for Children 69(1): 81-86, 13.
9. Dong, Y., et al. (2014). "Remineralization of early caries by chewing sugar-free gum: A clinical study using quantitative light-induced fluorescence." American Journal of Dentistry 27(6): 291-295.
10. Frost, P. M., et al. (2006). "Impact of wearing an intra-oral lubricating device on oral health in dry mouth patients." Oral Diseases 12(1): 57-62.
11. Lif Holgerson, P., et al. (2005). "Effect of xylitol-containing chewing gums on interdental plaque-pH in habitual xylitol consumers." Acta odontologica Scandinavica 63(4): 233-238.
12. Masoud, M. I., et al. (2015). "Long-term clinical and bacterial effects of xylitol on patients with fixed orthodontic appliances." Progress in Orthodontics 16: 35.
13. Simons, D., et al. (2002). "The effect of medicated chewing gums on oral health in frail older people: a 1-year clinical trial." Journal of the American Geriatrics Society 50(8): 1348-1353.
14. Jensen, J. L., et al. (1998). "Salivary secretion: stimulatory effects of chewing-gum versus paraffin tablets." European journal of oral sciences 106(4): 892-896.
15. Dawes, C. and Kubieniec, K., 2004. The effects of prolonged gum chewing on salivary flow rate and composition. Archives of Oral Biology, 49(8), pp.665-669. (From: Cai, 2009)
16. Thabuis, C., et al. (2013). "Effects of maltitol and xylitol chewing-gums on parameters involved in dental caries development." European Journal of Paediatric Dentistry 14(4): 303-308
17. Ly, K. A., et al. (2006). "Linear response of mutans streptococci to increasing frequency of xylitol chewing gum use: A randomized controlled trial [ISRCTN43479664]." BMC Oral Health 6.2010
18. Polland, K. E., et al. (2003). "Salivary flow rate and pH during prolonged gum chewing in humans." Journal of Oral Rehabilitation 30(9): 861-865.

# D. Birkhed, S. Edwardsson, U. Wikesjö, Marie-Louise Ahlden, J. Ainamo. 1983. Effect of 4 Days Consumption of Chewing Gum Containing Sorbitol or a Mixture of Sorbitol and Xylitol on Dental Plaque and Saliva. Caries Res. 17: 76-88 (1983)

1. Barmes, D., et al. (1985). "Field trials of preventive regimens in Thailand and French Polynesia." International Dental Journal 35(1): 66-72.
2. Hoerman, K. C., et al. (1990). "Effect of gum chewing on plaque accumulation." Journal of Clinical Dentistry 2(1): 17-21.
3. Massoth, D., et al. (2006). "The effect of xylitol on Streptococcus mutans in children." Journal of the California Dental Association 34(3): 231-234.
4. Loesche, W. J., et al. (1984). "The effect of chewing xylitol gum on the plaque and saliva levels of Streptococcus mutans." Journal of the American Dental Association 108(4): 587-592.
5. Alanen, P., et al. (2000). "Xylitol candies in caries prevention: results of a field study in Estonian children." Community Dentistry & Oral Epidemiology 28(3): 218-224.
6. Isokangas, P., et al. (1989). "Long‐term effect of xylitol chewing gum on dental caries." Community Dentistry and Oral Epidemiology 17(4): 200-203.
7. Tanaka, M., Toe, M., Nagata, H., Ojima, M., Kuboniwa, M., Shimizu, K., ... & Shizukuishi, S. (2010). Effect of Eucalyptus‐Extract Chewing Gum on Oral Malodor: A Double‐Masked, Randomized Trial. Journal of periodontology, 81(11), 1564-1571.
8. Soderling, E., et al. (2000). "Influence of maternal xylitol consumption on acquisition of mutans streptococci by infants." Journal of Dental Research 79(3): 882-887.
9. Makinen, K. K., et al. (1996). "Polyol chewing gums and caries rates in primary dentition: a 24-month cohort study." Caries Research 30(6): 408-417.
10. Makinen, K. K., et al. (2008). "Thirty-nine-month xylitol chewing-gum programme in initially 8-year-old school children: a feasibility study focusing on mutans streptococci and lactobacilli." International Dental Journal 58(1): 41-50.
11. Soderling E, Makinen,KK, Chen C-Y, Pape HR, Loesche W, Makinen P-L Effects of sorbitol, xylitol, and xylitol-sorbitol chewing gums on dental plaque. Caries Res 189;23:378-84
12. Aluckal, E. and A. V. Ankola (2018). "Effectiveness of xylitol and polyol chewing gum on salivary streptococcus mutans in children: A randomized controlled trial." Indian journal of dental research : official publication of Indian Society for Dental Research29(4): 445-449.
13. Keijser, B. J. F., et al. (2018). "The Impact of Maltitol-Sweetened Chewing Gum on the Dental Plaque Biofilm Microbiota Composition." Frontiers in Microbiology9: 381.
14. Cocco, F., et al. (2020). "Concentration in saliva and antibacterial effect of Xylitol chewing gum: In vivo and in vitro study." Applied Sciences (Switzerland)10(8).
15. Dehghanmehr, S., et al. (2018). ??IT IS DRY MOUTH BUT NOT FOR ORAL HEALTH REASONS PER SAY

"Investigating the impact of sugar free gum on the thirst and dry mouth of patients undergoing hemodialysis." International Journal of Pharmaceutical Sciences and Research9(5): 2062-2066.

1. Lus, G., et al. (2018). TYPE OF SFG? OUTCOME RELEVANT? "Palatability and oral cavity tolerability of THC:CBDoromucosal spray and possible improvement measures in multiple sclerosis patients with resistant spasticity: a pilot study." Neurodegenerative Disease Management8(2): 105-113.
2. Manisha, et al. (2019). TYPE OF SFG "Comparison of the effect of salt water rinse, sugar-free chewing gum and tooth brushing on the pH of saliva." International journal of research in pharmaceutical sciences10(2): 922‐926.
3. Utami, K. C., et al. (2018). UNSURE OF SFG INGREDIENT OR OUTCOME RELEVANCE "Chewing gum is more effective than saline-solution gargling for reducing oral mucositis." EnfermeriaClinica28(Suppl 1): 5‐8.

- Language N= 15

1. Buhmann, A. G., et al. (1991). "Tooth protecting chewing gum tablets for lessening caries risk." Oral-prophylaxe / Herausgeber, Verein fur Zahnhygiene e.V 13(4): 142-147.
2. Hase, J. C., et al. (1992). "An individual training-program for speeding up prolonged oral sugar clearance in hospitalized elderly patients - a pilot-study." Swedish dental journal 16(6): 239-245.
3. Li, X. J., et al. (2010). "[Comparative effects of the maltitol chewing gums on reducing plaque]." Hua Xi Kou Qiang Yi Xue Za Zhi 28(5): 502-504.
4. Porciani, P. F., et al. (1992). "[The action on bacterial plaque of the mastication of a sorbitol chewing gum]." Minerva Stomatologica 41(10): 445-449.
5. Rekola, M. (1989). "[Effect of Xylitol chewing gum on total saliva and dental plaque in caries-active persons]." Oral-Prophylaxe 11(3): 95-100.
6. Rekola, M. (1986). "[Incipient carious lesion changes as effected by the use of xylitol]." Proceedings of the Finnish Dental Society 82(1): 39-41.
7. Rumiantsev, V. A., et al. (2011). "[Sugar of substitute stevioside in chewing gum: comparative double blind controllable study]." Stomatologiia 90(1): 18-21.
8. Schneider, P. and H. R. Muhlemann (1976). "[Sugar-free, tooth-protecting chewing gum and candy. Results of a 7-year study]." SSO: Schweizerische Monatsschrift fur Zahnheilkunde 86(2): 150-166.
9. Maiwald, H. J., et al. (1982). "[Effect of sugared and sugarfree chewing gum on plaque pH]." Zahn-, Mund-, und Kieferheilkunde Mit Zentralblatt 70(6): 598-604. [GERMAN]
10. Makinen, K. K. and A. Scheinin (1975). "[Sugar studies in Abo. Report of clinical long-range studies]." Tandlakartidningen 67(1): 8-20. [SWEDEN]
11. Kertesz, P., et al. (1988). "[3 years' results of the WHO xylitol caries-preventing program in Hungarian children's homes. VI. Changes in the carbohydrate-protein ratio in dental plaque]." Fogorvosi Szemle 81(2): 33-37. [HUNGARIAN]
12. Tsapok, P. I., et al. (2012). "[Sugar substitute products impact on oral fluid biochemical properties]." Stomatologiia 91(2): 23-25.
13. Szoke, J., et al. (2002). "[Effect of chewing sugar-free gum on dental caries]." Fogorvosi Szemle 95(1): 21-25.
14. Scheinin, A. and K. K. Makinen (1977). "Effect of consumption of certain sugars on the incidence of dental caries in man." Czasopismo Stomatologisczne 30(6): 481-489.
15. Shu, C., et al. (2007). "Effect of two kinds of chewing gums on dental plaque pH." Shanghai kou qiang yi xue = Shanghai journal of stomatology 16(2): 172-175.

- Duplicates N=20

1. Nagata, H., Inagaki, Y., Tanaka, M., Ojima, M., Kataoka, K., Kuboniwa, M., ... & Shizukuishi, S. (2008). Effect of Eucalyptus Extract Chewing Gum on Periodontal Health: A Double‐Masked, Randomized Trial. Journal of periodontology, 79(8), 1378-1385.
2. Kandelman, D. and G. Gagnon (1987). "Clinical results after 12 months from a study of the incidence and progression of dental caries in relation to consumption of chewing-gum containing xylitol in school preventive programs." Journal of Dental Research 66(8): 1407-1411. (From: Kandelman, 1990)
3. Jagodzinska, M., et al. (2011). "Three months of regular gum chewing neither alleviates xerostomia nor reduces overhydration in chronic hemodialysis patients." Journal of Renal Nutrition 21(5): 410-417.
4. Said, H. and H. Mohammed (2013). "Effect of chewing gum on xerostomia, thirst and interdialytic weight gain in patients on hemodialysis." Life Science Journal 10(2): 1767-1777.
5. Soderling, E., et al. (1997). "Effects of xylitol, xylitol-sorbitol, and placebo chewing gums on the plaque of habitual xylitol consumers." European journal of oral sciences 105(2): 170-177.
6. Scheinin, A. (1976). "Caries control through the use of sugar substitutes." International Dental Journal 26(1): 4-13.
7. Scheinin, A. (1976). "Xylitol in relation to the incidence of dental caries." Internationale Zeitschrift fur Vitamin- und Ernahrungsforschung - Beiheft15: 358-367
8. Tellefsen, G., et al. (1996). "Use of chlorhexidine chewing gum significantly reduces dental plaque formation compared to use of similar xylitol and sorbitol products." Journal of Periodontology 67(3): 181-183.
9. Steinberg, L. M., et al. (1992). "Remineralizing potential, antiplaque and antigingivitis effects of xylitol and sorbitol sweetened chewing gum." Clinical Preventive Dentistry 14(5): 31-34.
10. Waaler, S. M., et al. (1993). "Adaptation of dental plaque to sorbitol after 3 months' exposure to chewing gum." European journal of oral sciences 101(2): 84-86.
11. Waler, S. M., et al. (1992). "Xylitol 5-P formation by dental plaque after 12 weeks' exposure to a xylitol/sorbitol containing chewing gum." Scandinavian Journal of Dental Research 100(6): 319-321.
12. Holgerson, P. L., et al. (2007). "Decreased salivary uptake of [14C]-xylitol after a four-week xylitol chewing gum regimen.[Erratum appears in Oral Health Prev Dent. 2008;6(1):81]." Oral Health & Preventive Dentistry 5(4): 313-319.
13. Rekola, M. (1982). "A comparison of the effects of xylitol and sorbitol sweetened chewing gums on dental plaque." Proceedings of the Finnish Dental Society 78(3): 128-133.
14. Rekola, M. (1989). "Correlation between caries incidence and frequency of chewing gum sweetened with sucrose or xylitol." Proceedings of the Finnish Dental Society 85(1): 21-24.
15. Twetman, S. and C. Stecksen-Blicks (2003). "Effect of xylitol-containing chewing gums on lactic acid production in dental plaque from caries active pre-school children." Oral Health & Preventive Dentistry 1(3): 195-199.
16. Isokangas, P., et al. (1988). "Xylitol chewing gum in caries prevention: a field study in children." Journal of the American Dental Association 117(2): 315-320.
17. Mäkinen, K. K. (1996). "Polyol-combinant saliva stimulants and oral health in Veterans Affairs patients - An exploratory study." Special Care in Dentistry 16(3): 104-115.
18. Soderling, E., et al. (1989). "Effect of sorbitol, xylitol, and xylitol/sorbitol chewing gums on dental plaque." Caries Research 23(5): 378-384.
19. Takahashi, K., Fukazawa, M., Motohira, H., Ochiai, HOERMK., Nishikawa, H., & Miyata, T. (2003). A pilot study on antiplaque effects of mastic chewing gum in the oral cavity. Journal of periodontology, 74(4), 501-505.
20. Rekola, M. (1986). "A planimetric evaluation of approximal caries progression during one year of consuming sucrose and xylitol chewing gums." Proceedings of the Finnish Dental Society 82(4): 213-218.
